# Hyponatremia and risk factors for death in human visceral leishmaniasis: new insights from a cross-sectional study in Brazil

**DOI:** 10.1186/s12879-017-2257-4

**Published:** 2017-02-23

**Authors:** Elizabeth De Francesco Daher, Douglas de Sousa Soares, Sérgio Luiz Arruda Parente Filho, Gdayllon Cavalcante Meneses, Tainá Veras de Sandes Freitas, Tacyano Tavares Leite, Geraldo Bezerra da Silva Junior

**Affiliations:** 1 Department of Internal Medicine, School of Medicine, Federal University of Ceará, Rua Vicente Linhares, 1198, Fortaleza, CE CEP: 60270-135 Brazil; 2 Pharmacology Graduate Program, Department of Physiology and Pharmacology, School of Medicine, Federal University of Ceará, Fortaleza, Ceará Brazil; 3 School of Medicine, Public Health Graduate Program, Health Sciences Center, University of Fortaleza, Fortaleza, Ceará Brazil; 4 Medical Sciences Graduate Program, Department of Internal Medicine, School of Medicine, Federal University of Ceará, Rua Vicente Linhares, 1198, Fortaleza, CE CEP: 60270-135 Brazil

**Keywords:** Visceral leishmaniasis, Kala-azar, Predictor factors, Hypoalbuminemia, Hyponatremia, Mortality

## Abstract

**Background:**

Visceral leishmaniasis (VL) is an important and potentially fatal neglected tropical disease. The aim of this study was to investigate hyponatremia and risk factors for death among VL patients.

**Methods:**

This is a cross-sectional study with VL patients admitted to a tertiary hospital in Northeast Brazil, from 2002 to 2009. Patients were divided into two groups: non-survivors and survivors. Hyponatremia was defined as serum sodium < 135 mEq/L. A logistic regression model was done to investigate risk factors for death.

**Results:**

A total of 285 VL patients were included, with mean age 37 ± 15 years, and 74% were males. Thirty-four patients died (11.9%). Non-survivors had a significantly higher prevalence of dyspnea (38.2 vs. 16.7%, *p* = 0.003), pulmonary crackles (11.8 vs. 4.0%, *p* = 0.049), dehydration (23.5 vs. 10.8%, *p* = 0.033), oliguria (8.8 vs. 0.8%, *p* = 0.001) and jaundice (47.1 vs. 14.3%, *p* < 0.001). They also presented higher prevalence of hyponatremia (41.9 vs. 24.1%, *p* = 0.035), thrombocytopenia (91.2 vs. 65.3%, *p* = 0.002) and severe hypoalbuminemia (78.3 vs. 35.3%, *p* < 0.001). In multivariate analysis, moderate/severe hyponatremia (OR = 2.278, 95% CI = 1.046–4.962), thrombocytopenia (OR = 5.482, 95% CI = 1.629–18.443), jaundice (OR = 5.133, 95% CI = 1.793–14.696) and severe hypoalbuminemia (OR = 6.479, 95% CI = 2.124–19.766) were predictors of death.

**Conclusion:**

Higher prevalence of dehydration, oliguria, pulmonary symptoms and liver involvement was found in non-survivors VL patients. Hypoalbuminemia and hyponatremia were frequent and significantly associated with mortality.

## Background

Visceral Leishmaniasis (VL), also known as kala-azar, is a zoonosis caused by protozoan parasites from the genus *Leishmania*, which are typically found in tropical areas. The disease is endemic in many countries, including Brazil, which responds for 90% of cases in the Americas [1]. Due to its high incidence and the potential harms caused by the illness, it has become one of the World Health Organization’s (WHO) top priorities among neglected tropical diseases [2].

The *Leishmania* parasites escape the hosts’ immune response and cause a severe immune dysfunction, with polyclonal activation of B lymphocytes as well as cellular and humoral immunity impairment. During the progression of the disease, the parasites affect reticuloendothelial tissues and important organs, such as lymph nodes, bone marrow, liver, spleen, digestive system and kidneys [3]. This condition predominantly affects children and young adults, and may manifest as an oligosymptomatic infection with non-specific symptoms such as low fever, dry coughing, sweating and malaise, or as a life-threatening chronic condition with pancytopenia, hemorrhagic phenomena, hepatomegaly, splenomagly, hypoalbuminemia and elevated globulinemia [4, 5].

Unfortunately, mortality from VL is still unacceptable high, reaching 95% when untreated [5]. There are some studies investigating factors associated with poor outcome in VL. A recent study from India evidenced some epidemiologic aspects as associated with less mortality in VL: treatment at public facility, shorter (≤30 days) diagnostic delay and treatment completion [6]. Other studies found some clinical aspects as risk factors for death in VL, such as: secondary bacterial infections, edema, jaundice, thrombocytopenia, hemorrhage, diarrhea, age (young children or elderly), severe neutropenia, dyspnea, and hypoalbuminemia, among others [7–10].

Hence, the aim of this study was to investigate hyponatremia and risk factors for death among VL patients.

## Methods

### Studied population

This is a cross-sectional study conducted with VL patients consecutively admitted to the São José Infectious Diseases hospital in Fortaleza, Ceará, Brazil, from January 2002 to December 2009. All VL cases admitted in the study period were included. This hospital is a reference for all infectious diseases in the state of Ceará, Northeast Brazil, which is endemic for VL. All patients had clinical and epidemiological data suggestive of VL. Diagnosis was confirmed by immunofluorescence, reactive in serology test using K39 antigen or the presence of amastigote forms of the parasite in the bone marrow smears. Inclusion criteria were: VL confirmed diagnosis and age ≥ 15 years, due to differences in laboratory and acute kidney injury (AKI) definition for the pediatric population.

In order to perform a comparison, patients were divided into two different groups: non-survivors (included patients who died during hospitalization) and survivors (included the patients who did not).

### Treatment protocol

According to the treatment protocol from the Brazilian Ministry of Health, patients were treated with pentavalent antimonial (meglumine antimoniate - Glucantime®) 20 mg/kg daily for 20 to 40 consecutive days. In cases of unsuccessful treatment with pentavalent antimonial or toxicity to this drug, amphotericin B (0.7 – 1.0/kg) daily for 14 to 21 consecutive days was indicated. However, amphotericin B was indicated as the first line drug in some cases: deteriorated clinical status, age > 50 years, QT interval > 450 ms on electrocardiography or use of medication which enlarge QT interval, allergy to pentavalent antimonial, immune deficiencies or use of immune suppressors and pregnant women. The formulation of amphotericin B used (liposomal or deoxycholate) depended on drug availability during hospitalization. None of the researchers interfered with drug choice.

Clinical and demographical parameters included age, gender, hospitalization time, time between onset of symptoms and hospitalization, main signs and symptoms on admission, treatments and AKI development. Treatment parameters included a record of all VL drug therapies initiated after hospital admission.

### Biochemical evaluation

Biochemical analyses were performed using a routine automated analyzer (Cobas C111, Roche®), including serum urea and creatinine, albumin, globulins, aspartate amino transaminase (AST), alanine amino transaminase (ALT), total bilirubin, indirect bilirubin, direct bilirubin.

Serum urea in samples was hydrolyzed by urease to form ammonia that reacts with alfa-ketoglutarate and NADH in the presence of GLDH enzyme. The decrease in absorbance due to consumption of NADH is proportional to urea levels. Serum creatinine was quantified by colorimetric assay based on the Jaffé method. In alkaline solution, creatinine forms a yellow-red complex with picrate. The rate of dye formation is proportional to the creatinine concentration in the specimen. Also, serum albumin, globulins, AST, ALT, total, direct and indirect bilirubin were determined by colorimetric assay.

Sodium and potassium were determined by ion selective electrolyte analyzer (9180, Electrolyte Analyzer, Roche®) and expressed as mEq/L.

Hemoglobin (Hb), hematocrit, leukocytes count, platelets count were determined by Automated HematologyDiagnostics (Sysmex® XT4000i). Arterial blood gas analysis included pH, arterial carbon dioxide partial pressure (pCO_2_) and bicarbonate (HCO_3_) were determined through “Blood gas analyzer” machine (Stat Profile Prime® Critical Care Analyser).

### Definitions

AKI was defined according to Kidney Diseases Improving Global Outcomes (KDIGO) criteria [11]. Hypotension was defined as mean arterial blood pressure (MAP) < 60 mmHg, and therapy with vasopressors was initiated when the MAP remained < 60 mmHg despite of fluid administration. Oliguria was defined as urine output < 0.5 mL/kg/h after adequate fluid replacement.

Hyponatremia was defined as serum sodium (Na^+^) < 135 mEq/L. Patients were classified among hyponatremia severity: mild hyponatremia was defined as Na^+^ between 134 and 131 mEq/L, moderate hyponatremia as Na^+^ between 130 and 126 mEq/L and severe hyponatremia as Na^+^ ≤ 125 mEq/L. Hypokalemia was defined as serum potassium < 3.5 mEq/L. Anemia was defined as Hb < 13.0 g/dL in males and Hb < 12.0 g/dL in females. Leukopenia was defined as leukocytes < 4,000/mm^3^ and thrombocytopenia as platelets < 100,000/mm^3^. Hypoalbuminemia was defined as serum albumin < 3.5 g/dL and severe hypoalbuminemia as serum albumin < 2.5 g/dL.

### Statistical analysis

Statistical analysis was executed using SPSS program for windows version 20.0 (IBM, Chicago – IL, USA) and its results were expressed through tables. Kolmogorov-Smirnov test was used for numeric variables, in order to assess variable distribution. Variables with normal distribution were expressed through mean ± standard deviation (SD). Variables with non-normal distribution were expressed through median values. Patients were divided into two groups in order to perform a comparison: those patients who died (non-survivors) and those who did not (survivors). Comparison of categorical variables was executed using Pearson’s Chi-square while numerical variables were compared using Student’s *T* test (for variables with normal distribution) or Mann-Whitney test (for variables with non-normal distribution). *P* values ≤ 0.05 were considered statistically significant. In order to evaluate risk factors or protective factors for death, a logistic regression model was used for categorical variables. Adjusted odds ratios (OR) and 95% confidence intervals (CI) were calculated. All variables which presented statistical significance (*p* ≤ 0.05) in the univariate analysis were included in the logistic regression.

## Results

In the studied period, 285 patients with VL were admitted, with mean age of 37 ± 15.3 years, and 74% were males. Among them, 34 died (11.9%). There was not any difference between survivors and non-survivors regarding age, gender, hospitalization time or time between onset of symptoms and hospitalization, as summarized in Table 1.

**Table 1:** Comparison of demographic data between survivors and non-survivors patients with visceral leishmaniasis

	Non-survivors (*N* = 34)	Survivors (*N* = 251)	*p*
Age (years)	42.8 ± 19.5	36.3 ± 14.6	0.067
Gender			
*Male*	26 (76.5%)	185 (77.7%)	0.873
*Female*	8 (23.5%)	56 (22.3%)	
Time between onset of symptoms and admission (days)	94 (5–730)	87 (1–720)	0.422
Hospitalization time (days)	21 (2–66)	20 (2–221)	0.469

Values were represented as mean ± SD, median (range) or percentages. Student’s *T* test and Chi-squared tests were used. *P* ≤ 0.05 was considered statistically significant

Regarding treatment, the majority of patients were exclusively treated with pentavalent antimonial (68.2%). Seventy-seven patients (27%) used deoxycholate amphotericin B and 14 (4.8%) used liposomal amphotericin B. Frequency of exclusive pentavalent antimonial treatment was significantly lower in non-survivors than survivors (50.0% vs. 70.5%, *p* = 0.003), but pentavalent antimonial use was not a protective factor for death in multivariate analysis.

Non-survivors presented a significantly higher prevalence of vomiting (38.2 vs. 21.5%, *p* = 0.031), dehydration (23.5 vs. 10.8%, *p* = 0.033), edema (32.4 vs. 16.7%, *p* = 0.028), oliguria (8.8 vs. 0.8%, *p* = 0.001), dyspnea (38.2 vs. 16.7%, *p* = 0.003), pulmonary crackles (11.8 vs. 4.0%, *p* = 0.049) and jaundice (47.1 vs. 14.3%, *p* < 0.001) on admission, as presented in Table 2. They also had higher prevalence of moderate hyponatremia (41.9 vs. 24.1%, *p* = 0.035), thrombocytopenia (91.2 vs. 65.3%, *p* = 0.002) and severe hypoalbuminemia (78.3 vs. 35.3%, *p* < 0.001).

**Table 2:** Comparison of main signs and symptoms between survivors and non-survivors patients with visceral leishmaniasis

	Non-survivors (*N* = 34)	Survivors (*N* = 251)	*p*
Fever	30 (88.2%)	229 (91.2%)	0.569
Hepato-splenomegaly	22 (64.7%)	199 (79.3%)	0.056
Weight loss	22 (64.7%)	185 (74%)	0.253
Asthenia	16 (47.1%)	156 (62.2%)	0.091
Cough	13 (38.2%)	95 (37.8%)	0.965
Abdominal pain	15 (44.1%)	84 (33.5%)	0.221
Diarrhea	12(35.3%)	57 (22.7%)	0.108
Vomiting	13 (38.2%)	54 (21.5%)	0.031
Dyspnea	13 (38.2%)	42 (16.7%)	0.003
Edema	11 (32.4%)	42 (16.7%)	0.028
Jaundice	16 (47.1%)	36 (14.3%)	<0.001
Dehydration	8 (23.5%)	27 (10.8%)	0.033
Nose bleeding	11 (4.8%)	13 (5.2%)	0.128
Pulmonary crackles	4 (11.8%)	10 (4.0%)	0.049
Oliguria	3 (8.8%)	2 (0.8%)	0.001
Hypotension	0 (0%)	2 (0.8%)	0.602

Chi-squared test was used. *P* ≤ 0.05 was considered statistically significant

Acute kidney injury (AKI) was observed in 93 patients (32.6%). Among AKI patients, most of them were in stage 2 (41.9%) or stage 3 (40.8%) according to KDIGO classification, but there was not significant differences between groups in AKI development.

In addition, non-survivors had significantly lower levels of hemoglobin (6.5 ± 1.8 vs. 7.5 ± 1.5 g/dL, *p* < 0.001), hematocrit (20.4 ± 5.0 vs. 23.1 ± 4.6%, *p* = 0.002), leukocytes count (3904 vs. 3688/mm^3^, *p* = 0.01), platelets count (32.0 ± 28.6 vs. 84.0 ± 62.1 10^3^/mm^3^, *p* = 0.017), serum sodium (130.6 ± 5.4 vs. 133.2 ± 4.2 mEq/L, *p* = 0.005) and serum albumin (2.04 ± 0.74 vs. 2.70 ± 0.79 g/dL, *p* < 0.001), as well as higher levels of AST (median: 295 vs. 104 IU/L, *p* = 0.039), total bilirubin (median: 7.0 vs. 1.3 mg/dL, *p* < 0.001), indirect bilirubin (median: 5.4 vs. 0.9 mg/dL, *p* < 0.001), direct bilirubin (median: 1.6 vs. 0.4 mg/dL, *p* = 0.004) and PCO_2_ (median: 36.7 ± 10.1 vs. 31.6 ± 6.2 mmHg, *p* = 0.044), as demonstrated in Table 3. There was not significant difference in serum urea, creatinine, potassium, globulins, ALT, arterial pH and bicarbonate.

**Table 3:** Comparison of laboratory data, at hospital admission, between survivors and non-survivors patients with visceral leishmaniasis

	Non-survivors (*N* = 34)	Survivors (*N* = 251)	*p*
Hemoglobin (g/dL)	6.5 ± 1.8	7.5 ± 1.5	< 0.001
Hematocrit (%)	20.4 ± 5.0	23.1 ± 4.6	0.002
Leukocytes (/mm^3^)	3904 (900–16,800)	3688 (300–11,000)	0.01
Platelets (10^3^/mm^3^)	32.0 ± 28.6	84.0 ± 62.1	0.017
Urea (mg/dL)	57.1 ± 37.9	51.8 ± 38.9	0.469
Creatinine (mg/dL)	1.07 ± 0.47	1.15 ± 1.13	0.750
Sodium (mEq/L)	130.6 ± 5.4	133.2 ± 4.2	0.005
Potassium (mEq/L)	4.2 ± 0.8	4.0 ± 0.6	0.297
Albumin (g/dL)	2.04 ± 0.74	2.70 ± 0.79	< 0.001
Globulins (g/dL)	4.26 ± 1.26	4.32 ± 1.65	0.915
AST (U/L)	295 (12–2161)	104 (13–1165)	0.039
ALT (U/L)	192 (5–1071)	78 (4–603)	0.065
Total bilirubin (mg/dL)	7.0 (1.0–22.3)	1.3 (0.04–21.1)	< 0.001
Direct Bilirubin (mg/dL)	5.4 (0.66–17.79)	0.9 (0.07–16.7)	< 0.001
Indirect Bilirubin (mg/dL)	1.6 (0.08–7.0)	0.4 (0.06–4.4)	0.04
Arterial pH	7.34 ± 0.14	7.44 ± 0.06	0.059
HCO_3_ (mEq/L)	16.7 ± 7.1	20.7 ± 6.5	0.178
PCO_2_ (mmHg)	36.7 ± 10.1	31.6 ± 6.2	0.044

*AST* aspartate aminotransferase, *ALT* alanine aminotransferase, *HCO3* serum bicarbonate, *PCO2* carbon dioxide partial pressure

Values were represented as mean ± SD or median (range). Student’s *T* test and Mann-Whitney tests were used. *P* ≤ 0.05 was considered statistically significant

Multivariate analysis showed that jaundice (*p* = 0.002, OR = 5.133, 95% CI = 1.793–14.696), thrombocytopenia (*p* = 0.006, OR = 5.482, 95% CI = 1.629–18.443), severe hypoalbuminemia (*p* = 0.001, OR = 6.479, 95% CI = 2.124–19.766) and moderate/severe hyponatremia (*p* = 0.038, OR = 2.278, 95% CI = 1.046–4.962) on admission were important risk factors for death, as evidenced in Table 4.

**Table 4:** Risk factors for death among survivors and non-survivors patients with visceral leishmaniasis

	OR	95% CI	*p*
Hypoalbuminemia (severe)	6.479	2.124–19.766	0.001
Thrombocytopenia	5.482	1.629–18.443	0.006
Jaundice	5.133	1.793–14.696	0.002
Hyponatremia (moderate/severe)	2.278	1.046–4.962	0.038

*OR* Odds Ratio, *CI* Confidence Interval

*P* ≤ 0.05 was considered statistically significant

## Discussion

VL is an important zoonotic disease which presents an alarmingly elevated prevalence in the Brazilian state of Ceará, where this research was conducted [1]. This disease presents a broad spectrum of symptoms, which may vary from an oligosymptomatic infection to a life-threatening chronic condition [5]. This is one of the few studies to investigate the role of hyponatremia in VL and the first to found a significant association of this disturbance with mortality in VL.

VL clinical manifestations are dependent on host’s immune response. When type I immune response is triggered, in general, there is effective protection against Leishmanias, due to activation of infected phagocytes, which leads to destruction of intracellular amastigotes [12]. Disease can progress due to multiple factors that block adequate response, including the action of suppressive cytokines, exhaustion of specific T cells, loss of lymphoid tissue architecture and a defective humoral response [12]. In the present study it was not possible to assess patients’ immune response because this is not routine in our hospital.

The patients in our study were mostly treated with pentavalent antimonials (PA), according to treatment protocol from Brazilian Ministry of Health. This type of drug has well known adverse effects, such as cardiac, renal and pancreatic toxicities. It enters the host cells and kills amastigote forms of the parasite, but also induces DNA toxicity and cellular necrosis in the human host [13, 14]. In the present study, non-survivors used significantly less PA than survivors. Differently from what was found in previous studies, PA use and its side-effects were not associated with increased mortality risk in our cohort [15]. Patients who needed a second line drug (amphotericin B) were those who had contraindications to PA or presented a poorer clinical status, leading to higher propensity for mortality.

Regarding symptoms, non-survivors presented vomiting, dehydration and oliguria more frequently, reflecting the volume depletion they manifested on admission. However, it did not lead to important hemodynamic instability, since most of the patients did not present hypotension or other cardiovascular abnormality. This hypovolemic state may have contributed to hyponatremia development, due to vasopressin secretion, and stimulus of renin-angiotensin-aldosterone system, as suggested by others [3].

In addition, non-survivors manifested higher prevalence of pulmonary symptoms on admission, such as dyspnea and pulmonary crackles, as well as significantly higher CO_2_ partial pressure on arterial blood gas analysis. Lung involvement in VL has been previously noticed, usually persisting from beginning through the entire course of the disease. It usually presents as interstitial pneumonitis, but also may manifest as pleural effusions or secondary lobar pneumonia [16]. In a study with 50 children with VL, secondary bacterial lobar pneumonia was diagnosed in 10% of cases [17]. Studies have demonstrated that both mononuclear infiltrates in the interstitial space and B lymphocytes activation with Th2 immune pattern response may be responsible for VL pneumonitis [18, 19]. Interstitial pneumonitis has also been associated to syndrome of inappropriate antidiuretic hormone secretion (SIADH) development in VL patients, resulting in hyponatremia [3].

Interestingly, AKI prevalence was high, but there was not significant differences between groups, even though renal failure have been previously described as risk factor for death in VL patients [20]. Renal involvement in VL is usually mild and transitory, but loss of kidney function may happen in some cases [5, 21]. However, the majority of our patients presented moderate to severe forms of AKI. We believe that use of nephrotoxic VL treatments may have been key factors on AKI development instead of hemodynamic factors, leading to no differences between groups.

Jaundice, hypoalbuminemia and hyponatremia were significantly more prevalent in non-survivors, and they were risk factors for death in multivariate analysis. Jaundice may reflect both liver involvement in VL and hemolysis, since both direct and indirect bilirubin were elevated. Total bilirubin, alkaline phosphatase and AST were also significantly higher in non-survivors. Liver is one of main reticuloendothelial organs affected by kala-azar (others are spleen and bone marrow), due to the presence of mononuclear macrophage system cells, as well as hepatic stellar cells [22]. The most frequently observed form of hepatic involvement in VL is hepatomegaly with chronic elevation of transaminases and bilirubins, due to direct infection by the parasite as well as hepatotoxicity of some medications, but it may rarely presents as acute liver disease [23, 24]. VL causes several changes in the liver, which leads to mononuclear cell infiltration, hepatocyte degeneration and fibrosis [25, 26]. The occurrence of jaundice was the strongest risk factor for severity in VL patients in a recent meta-analysis [7]. Thrombocytopenia has been previously recognized as a factor associated to adverse outcomes. In an important Brazilian meta-analysis, thrombocytopenia was a strong predictor of death among VL patients, a risk that was intensified in patients with platelets < 50,000/mm^3^ [7]. Low platelet count may be attributed to splenic platelets sequestration and systemic inflammation caused by VL parasites [27, 28]. In a recent study, thrombocytopenia was associated to elevated levels of IL-27, IL-10 and IL-6 cytokines. These cytokines interfere with macrophage activity and cause an impairment of Th1 response, predisposing to *Leishmania* proliferation and higher risk of unfavorable outcomes [29].

Hypoalbuminemia was very prevalent among our patients, and 40.5% of them presented severe hypoalbuminemia (serum albumin < 2.5 g/dL). Hypoalbuminemia in VL derives from hepatic dysfunction, impaired albumin production and reduced intestinal absorption of proteins due to protein loosing enteropathy [30]. These findings corroborate data in the literature, since albumin has been described as a factor for VL severity. In a Brazilian meta-analysis, low levels of albumin and the presence of edema were risk factors for severity and death in VL patients [7]. In another cohort study with VL children, serum albumin levels < 2.5 g/dL on admission were risk factors for poor outcomes [8]. Hyponatremia can be considered as the most frequent electrolyte disturbance in hospitalized patients and also in VL [31]. It has been described in a large variety of clinical situations, including medication related, cardiac, renal, hepatic and auto-immune conditions, as well as post-surgical conditions [32–36]. Low sodium levels are remarkably prevalent in infectious diseases, including viral, fungal, bacterial and protozoan infection [31]. In VL, hyponatremia prevalence > 90% was reported before [37]. Despite of that, there are scarce data in the literature relating hyponatremia to VL and there are not previous studies that describe hyponatremia as risk factors for death in kala-azar.

**Fig. 1:**
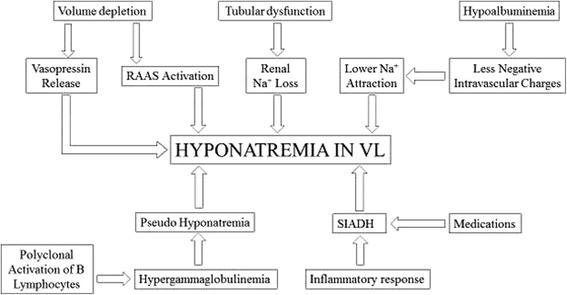
Pathophysiology of hyponatremia in visceral leishmaniasis (VL). *RAAS – renin-angiotensin-aldosterone system; Na^+^ - sodium; SIADH - syndrome of inappropriate antidiuretic hormone secretion; VL – visceral leishmaniasis

The pathogenesis of hyponatremia in VL is extremely complex and involves several hemodynamic and hormonal factors, as described in Fig. 1. Reduced body water may be considered the initial factor for hyponatremia in some patients, especially those who presented vomiting, diarrhea and clinical dehydration on admission. This volume depletion may lead to serum osmolality increase and vasopressin release, as well as renin-angiotensin-aldosterone system activation, leading to hyponatremia [1, 31]. Renal Na^+^ loss may also be considered a mechanism for hyponatremia. In a case series of 55 patients with VL, 15% of them had increased fractional excretion of sodium [36]. Tubular dysfunction is frequent in both visceral and cutaneous leishmaniasis which can contribute to hyponatremia development [38, 39]. Hypergammaglobulinemia, due to polyclonal activation of B lymphocytes, may also have contributed for hyponatremia. Gamma globulins occupy the space in serum volume, leading to lower readings of sodium and free water in serum plasma, a phenomenon called pseudo hyponatremia [31].

On the other hand, for patients who did not present dehydration on admission, hyponatremia may result from syndrome of inappropriate antidiuretic hormone secretion (SIADH), which is very common among VL patients [3]. This syndrome is characterized by hyponatremia and hypo-osmolality in normovolemic or hypervolemic patients. These patients usually present edema, as a result of total body water expansion, and preserve their renal water reabsorption mechanism [40, 41]. SIADH may be caused by medications, but most likely derives from the intense inflammatory response generated by multiple organ involvement in VL, which leads to activation of hypothalamic-pituitary axis and antidiuretic hormone release [42, 43]. Severe hypoalbuminemia, a condition very prevalent among our patients, may have also aggravated edema and contributed to hyponatremia development. Low serum albumin reduces plasma colloidosmotic pressure and reduces intravascular negative charges, inducing lower sodium attraction to intravascular space [3].

## Conclusions

Higher prevalence of dehydration, oliguria, pulmonary symptoms and liver involvement is found in non-survivors VL patients. They manifested higher rates of jaundice, thrombocytopenia, hypoalbuminemia and hyponatremia, which were the main predictors of death. Hyponatremia was frequent and significantly associated with mortality. Pathogenesis of hyponatremia in VL is multifactorial and involves both hypovolemic and hypervolemic conditions, as well as hypoalbuminemia and systemic inflammation. Renal tubular dysfunction may also play a role in VL-associated hyponatremia.

### Study limitations

The main limitations of this study derive from its retrospective nature. Patients’ data were collected from medical charts, and we unfortunately did not have access to some of patients’ information. The significant difference in the number of patients in each group may be considered a limitation, since it makes statistical analysis more difficult. However, we have chosen to compare these groups in order to investigate factors associated to mortality. Further studies are required to fully understand the pathophysiology of VL-associated hyponatremia and the best ways to prevent and treat this condition.

## Abbreviations

AKI: Acute kidney injury
ALT: Alanine amino transaminase
AST: Aspartate amino transaminase
CI: Confidence interval
Hb: Hemoglobin
HCO_3_: Bicarbonate
KDIGO: Kidney diseases improving global outcomes
MAP: Mean arterial blood pressure
Na^+^: Sodium
OR: Odds ratio
PA: Pentavalent antimonial
pCO_2_: Arterial carbon dioxide partial pressure
SD: Standard deviation
SIADH: Inappropriate antidiuretic hormone secretion
SPSS: Statistical package for social sciences
VL: Visceral leishmaniasis
WHO: World Health Organization.
